# An intercalation-type Li-free cathode with energy density exceeding 550 Wh kg^−1^

**DOI:** 10.1093/nsr/nwad032

**Published:** 2023-02-14

**Authors:** Kai Wu, Wenwei Luo, Bo Xu

**Affiliations:** Contemporary Amperex Technology Limited (CATL), China; Department of Physics, Jiangxi Normal University, China; Contemporary Amperex Technology Limited (CATL), China; Department of Physics, Jiangxi Normal University, China

The development of all-solid-state technology involving Li-free transition-metal-based cathodes and Li-metal anodes has become an emerging trend with regard to solving the energy and safety bottleneck of current Li-ion batteries [1,2]. Although relevant publications in this area have increased in recent years, most of them have focused on developing solid-state electrolytes and reviving Li-metal anodes, whereas much less attention has been paid to the customization of cathodes, which determine the overall cell energy density.

Intercalation-type Li-free cathodes storing guest ions in topotactic manners can effectively avoid drastic changes in micro/macrostructures and physical properties, achieving generally strong reversibility during the electrochemical process [3–5]. Exploring intercalation-type Li-free cathodes is expected to solve the long-standing open problem of large interfacial resistance between solid-state electrolytes and cathodes from the electrode design perspective. More importantly, it can also address concerns over raw material availability in Li-ion battery production [6]. Specifically, commercial cathodes suitable for large-scale energy storage applications (e.g. electric vehicles) that demonstrate high energy density and a long lifetime are highly reliant on Co or Ni, which is concerning owing to their high cost, scarcity and centralized/volatile supply chains. Therefore, the development and commercialization of Li-free cathodes without Co/Ni is critical for both the all-solid-state and traditional Li-ion battery industries.

However, the intrinsic voltage shortages of Li-free cathodes greatly limit their energy densities. Traditional regulation strategies (e.g. electronegativity, coordination and induction effect) based on changing the transition metal–ligand ionic/covalent properties are complex in terms of their mechanisms and with these it is technically difficult to achieve a direct enhancement in voltage [7,8]. Endowing Li-free cathodes with high energy densities comparable to those of traditional Li-containing cathodes (>550 Wh kg^−1^) while ensuring thermodynamic stability and interfacial compatibility with solid-state electrolytes remains a great challenge.

**Figure 1.**
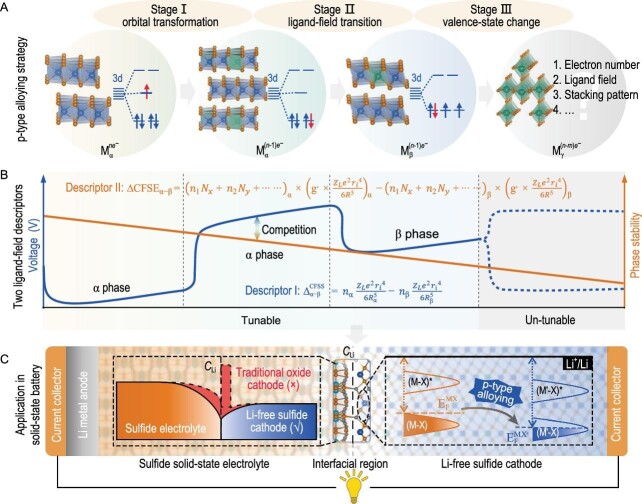
(A–C) Establishing a theoretical framework for quantitatively regulating voltage and phase stability competition based on a p-type alloying strategy combined with two improved ligand-field descriptors ($\Delta _{{{\alpha }} - {{\beta }}}^{{\rm{CFSS}}}{\rm{\ and\ }}\Delta {\rm{CFS}}{{\rm{E}}}_{{{\alpha }} - {{\beta }}}$). Reprinted with permission from ref. [9].

Recently, Siqi Shi's group from Shanghai University constructed a quantitative analysis on the critical voltage-tuning vs. phase-stability competition that has been overlooked in cathode systems [9]. Specifically, they proposed a p-type alloying strategy involving three interconnected stages (Fig. 1): molecular-orbital transformation, ligand-field transition and transition-metal valence-state change. Each regime was quantitatively characterized by the two improved ligand-field descriptors, viz., $\Delta _{{{\alpha }} - {{\beta }}}^{{\rm{CFSS}}}$ and ${\rm{CFS}}{{\rm{E}}}_{{{\alpha }} - {{\beta }}}$, which enable the adjusting of the voltage vs. phase stability balance to achieve an ideal voltage. Following this, a conceptually novel intercalation-type Li-free cathode, 2H-V_1.75_Cr_0.25_S_4_, was designed, which has a record energy density of >550 Wh kg^−1^ at electrode level, much higher than existing Li-free transition-metal-based electrodes (e.g. ∼500 Wh kg^−1^ for TiS_2_) and comparable to traditional Li-containing cathodes. Simultaneously, the design of such cathodes smoothed the Li^+^ distribution at the interface with solid-state electrolytes (such as Li_3_PS_4_), helping to address the interfacial compatibility challenges of oxide cathodes. Subsequently, 2H-V_1.75_Cr_0.25_S_4_ is obtained using a low temperature hydrothermal method and its superior voltage and energy density advantages are verified.

This work established a breakthrough understanding of the Fermi level and specific energy-density relationship of cathodes. The fundamental principle of the p-type alloying strategy may be generalized to a large family of charge-transfer-dominated ion-intercalation systems, which is both critical to all-solid-state battery science and cathode chemistry urgently addressing Co and Ni resource scarcities.
